# Upregulation of long noncoding RNAs PACER, MIAT, lincRNA-p21, and lincRNA-Cox2 in ulcerative colitis as potential biomarkers: A case-control study on their association with NF-кВ activation

**DOI:** 10.1097/MD.0000000000045219

**Published:** 2025-10-17

**Authors:** Amin Karimpour, Amir Anoshiravani, Mina Zare, Maryam Jabarpour, Farzad Farzamifar, Ghodratollah Panahi

**Affiliations:** a Department of Clinical Biochemistry, School of Medicine, Tehran University of Medical Sciences, Tehran, Iran; b Digestive Disease Research Center, Digestive Disease Research Institute, Tehran University of Medical Sciences, Tehran, Iran; c Department of Biochemistry, Shiraz University of Medical Sciences, Shiraz, Iran; d Department of Biochemistry, Faculty of Medicine, Tabriz University of Medical Sciences, Tabriz, Iran; e Department of Medical Genetics, School of Medicine, Tehran University of Medical Sciences, Tehran, Iran.

**Keywords:** IBD, inflammation, lncRNA, NF-κB, ulcerative colitis

## Abstract

Ulcerative colitis (UC), a subtype of inflammatory bowel disease, is characterized by chronic intestinal inflammation. The nuclear factor kappa B (NF-κB) pathway plays a critical role, and long noncoding RNAs (lncRNAs) are emerging as important regulators of gene expression. This study investigated 4 lncRNAs – PACER, myocardial infarction associated transcript (MIAT), lincRNA-p21, and lincRNA-Cox2–in colon tissues of UC patients and healthy controls, exploring their association with NF-κB activation. A total of 35 UC patients and 35 healthy controls were recruited from Shariati Hospital in Tehran, Iran. Real-time qPCR and Western blot measured lncRNA and NF-κB pathway protein levels. Statistical analyses included t-tests/Mann–Whitney U tests for group comparisons, Pearson/Spearman correlations for lncRNA-NF-κB relationships, and receiver operating characteristic analysis for diagnostic potential. PACER, MIAT, lincRNA-p21, and lincRNA-Cox2 were significantly upregulated in UC colon tissues compared to controls (all *P* ≤ .005). NF-κB p65 (*P* = .035) and phosphorylated NF-κB p65 (p-NF-κB p65; *P* = .008) levels were also significantly increased in UC. Expression of all 4 lncRNAs showed significant positive correlation with p-NF-κB p65 levels (all *P* < .05), suggesting a potential link between these lncRNAs and NF-κB activation in UC. receiver operating characteristic analysis demonstrated good diagnostic potential for these lncRNAs (AUCs > 0.91, all *P* < .0001) in differentiating UC. This study suggests that PACER, MIAT, lincRNA-p21, and lincRNA-Cox2 are dysregulated in UC and may be associated with NF-κB activation. These lncRNAs may serve as potential diagnostic biomarkers for UC. Further studies are needed to fully elucidate their role in UC pathogenesis and explore their potential as therapeutic targets.

## 1. Introduction

Ulcerative colitis (UC), a form of inflammatory bowel disease, is a chronic inflammatory condition characterized by persistent inflammation in the mucosal layer of the colon^[1]^ Common symptoms of UC include abdominal pain, bloody diarrhea, weakness, fatigue, and weight loss.^[2]^ While the prevalence of UC has stabilized in developed countries, it is on the rise in developing countries, posing a growing global health concern.^[3]^ A complex interplay of factors contributes to the development of UC, including genetic predisposition, gut microbiome composition, medication use, diet, sleep, stress, exercise, appendectomy, and smoking.^[4]^ These environmental and genetic factors can disrupt the delicate balance of immune responses in the digestive system, leading to chronic inflammation and damage to the mucosal layer of the colon.^[5]^

UC is characterized by chronic inflammation in the intestines, and numerous molecular pathways are involved in regulating this inflammation. One such pathway that plays a critical role in regulating inflammation and the pathogenesis of UC is the NF-κB pathway.^[6]^ Upon activation by inflammatory stimuli, the NF-κB pathway triggers the production of pro-inflammatory cytokines such as tumor necrosis factor-alpha (TNF-α), interleukin-1 (IL-1), and IL-6, which further regulate the expression of IL-12 and IL-23. These cytokines contribute to the chronic inflammation and tissue damage observed in UC.^[7]^ Consequently, the NF-κB pathway has garnered significant attention as a potential therapeutic target for UC, and extensive research is being conducted to explore strategies for modulating its activity to alleviate inflammation and improve disease outcomes.^[8–11]^ Furthermore, the chronic systemic inflammation characteristic of UC is known to induce a range of metabolic disturbances. Among these, alterations in serum lipid profiles, or dyslipidemia, have been reported, potentially reflecting the overall inflammatory burden and nutritional status of patients. Therefore, assessing these biochemical markers can provide a broader context for the pathophysiological changes occurring in UC.^[12]^

Long noncoding RNAs (lncRNAs) constitute a class of RNA molecules that, exceeding 200 nucleotides in length, lack protein-coding capacity.^[13]^ These RNAs perform diverse functions within the cell, notably the regulation of cellular signaling pathways.^[14]^ One such pathway is NF-κB, which plays a crucial role in modulating inflammatory responses. By regulating NF-κB activity, lncRNAs can influence the control of inflammation in various diseases, particularly UC. These lncRNAs participate in the disease process through diverse mechanisms, including the stimulation or inhibition of the NF-κB pathway.^[15,16]^

This study focuses on 4 specific lncRNAs: PTGS2 antisense NFKB1 complex-mediated expression regulator RNA (PACER), Homo sapiens myocardial infarction associated transcript (MIAT), Tumor protein p53 pathway corepressor 1 (lincRNA-p21), and prostaglandin-endoperoxide synthase 2, opposite strand 2 (lincRNA-Cox2). These lncRNAs have been previously implicated in the regulation of the NF-κB signaling pathway in various other diseases.^[17–19]^ Given the crucial role of NF-κB signaling in the pathogenesis of UC, it is plausible that these lncRNAs may also contribute to UC development and progression. However, their precise roles and expression patterns in the context of UC remain largely unknown. Therefore, this study aims to investigate the expression levels of PACER, MIAT, lincRNA-p21, and lincRNA-Cox2 in tissue samples from UC patients and healthy controls, and to explore the association between these expression levels and the degree of NF-κB pathway activation.

## 2. Materials and methods

### 2.1. Subjects

This study was approved by the Ethics Review Board of Tehran University of Medical Sciences (approval number IR.TUMS.DDRI.REC.1402.020), and written informed consent was obtained from all participants. This study included 70 participants: 35 patients diagnosed with UC and 35 individuals in a control group. The sample size of n = 35 participants per group was chosen based on sample sizes used in previous comparable studies investigating the expression of molecular markers in patients with UC.^[20,21]^ Participants were recruited from individuals who attended the Gastroenterology and Liver Clinic at Shariati Hospital in Tehran, Iran. The hospital is affiliated with Tehran University of Medical Sciences, and recruitment occurred between 2022 and 2024. Diagnosis of UC was based on comprehensive assessments, including clinical, laboratory, endoscopic, and histopathological findings. All participants were between 18 and 65 years old. The exclusion criteria were as follows: infectious enterocolitis, colorectal cancer, diabetes, heart disease, kidney disease, pregnancy, and polycystic ovary syndrome.

Tissue and serum samples were collected from all participants. Tissue samples were obtained by a gastroenterologist during colonoscopy procedures and immediately stored at -80°C. Serum samples were analyzed to determine levels of triglycerides (TG), total cholesterol, HDL cholesterol (HDL-C), and LDL cholesterol (LDL-C). Data collected from participants included age, sex, family history of UC, and medication history. Body mass index (BMI) was calculated using the standard formula: weight (kg)/ height (m)².

### 2.2. RNA extraction and cDNA synthesis

Total RNA was extracted from tissue samples using the AnaCell RNA Extraction Kit (AnaCell) according to the manufacturer’s protocol. cDNA synthesis was performed in 2 steps using the AnaCell cDNA Synthesis Kit (AnaCell), following the manufacturer’s instructions.

### 2.3. Quantitative real-time PCR

Quantitative real-time PCR (qPCR) was performed using RealQ Plus 2x Master Mix Green (Ampliqon) with 18S rRNA as an internal control. The following forward (F) and reverse (R) primers were used for PACER, MIAT, lincRNA-p21, lincRNA-Cox2 and 18s rRNA respectively: F:5’-CTTCTTCGCAGTCTTTGCCC-3’, R:5’-GGAGAGGAAGCCAAGTGTCC-3’, F:5’-GCAGATACAAGTGTGGAGTAAGC-3’, R:5’-ACAACCATCGCCAATCTCTATG-3’, F:5’-TGTTGCATTGTTGCATCATC-3’, R:5’-TTTCTTCCAGTGGTGAGTGG-3’, F:5’-CGGTGAAACTCTGGCTAGACAG-3’, R:5’-GCAAACCGTAGATGCTCAGGGA-3’, F:5’-CGGCGACGACCCATTCGAAC-3’, R:5’-GAATCGAACCCTGATTCCCCGTC-3’. qPCR was performed on a Rotor-Gene Q instrument (Qiagen) using 20 μL of master mix per reaction. The comparative CT method was used to determine the relative levels of target genes. All reactions were performed in duplicate to minimize technical variability.

### 2.4. Western blot

Colon tissues were homogenized in RIPA lysis buffer, and the supernatant containing protein was collected after centrifugation. Protein concentration was determined using the Bradford assay, with bovine serum albumin serving as the standard. Samples were separated by SDS-PAGE and transferred to a polyvinylidene difluoride (PVDF) membrane. The membrane was blocked with 2% nonfat dry milk in Tris-buffered saline containing Tween 20 (TBS-T) for 1 hour and 15 minutes at room temperature. It was then incubated with primary antibodies, followed by 3 washes using TBS-T, and subsequently incubated with horseradish peroxidase (HRP)-conjugated secondary anti-rabbit antibodies. Protein bands were detected using enhanced chemiluminescence (ECL) advanced reagents. The antibodies used in this study were listed as follows: NFκB p65 (F-6): sc-8008, p-NFκB p65 (27.Ser 536): sc-136,548, m-IgGκ BP-HRP: sc-516,102, mouse anti-rabbit IgG-HRP: sc-2357, GAPDH (0411): sc-47,724. Protein values were obtained through the ratio of target density/loading control (fold of control).

### 2.5. Statistical analyses

Statistical analyses were performed using GraphPad Prism (version 10.2.2) and IBM SPSS (version 27.0.1, Chicago). Data distribution was assessed for normality using the Shapiro–Wilk test. Normally distributed data are presented as mean ± SEM, while non-normally distributed data are presented as median [interquartile range (IQR)], with *P* < .05 considered statistically significant. For comparing 2 independent groups, an unpaired Student *t* test was applied to data with a normal distribution, while the Mann–Whitney U test was used for data with a non-normal distribution. Pearson correlation coefficient was employed to assess linear relationships between gene expression and biochemical indicators when both variables were normally distributed, whereas Spearman rank correlation coefficient was applied when one or both variables were not normally distributed. The diagnostic accuracy of gene biomarkers was evaluated using receiver operating characteristic (ROC) curve analysis, with the area under the curve (AUC) quantifying the ability of each biomarker to discriminate between groups.

## 3. Results

### 3.1. Demographic and biochemical characteristics of the subjects

This study included 35 individuals with UC and 35 healthy individuals as a control group. The age of participants ranged from 18 to 65 years. A significant difference was observed in the median age (*P* < .0001), BMI (*P* = .0192), and TG (*P* = .0040) between the 2 groups. However, no significant differences were found in the median levels of total cholesterol (*P* = .2201) and HDL-C (*P* = .0572), or in the mean levels of LDL-C (*P* = .7876). Additionally, the UC patient group was receiving various medications at the time of the study. A complete summary of these results is presented in Table 1.

**Table 1:** Clinical and biochemical characteristics of the study population.

Parameter	Control samples (n = 35)	UC samples (n = 35)	*P*-value
Age (year)	55 (28)	35 (18)	.0001
Gender (M/F)	11/24	17/18	.143
BMI (kg/m^2^)	24.69 (7.04)	22.60 (6.11)	.0192
TG (mg/dL)	162.5 (115.7)	101.5 (46.5)	.0040
Total cholesterol (mg/dL)	159.0 (23.8)	141.0 (34.2)	.2201
HDL‐C (mg/dL)	45.00 (12.75)	38.50 (14.75)	.0572
LDL‐C (mg/dL)	80.89 ± 8.729	77.85 ± 6.976	.7876
Smoking (n)	8/27	4/31	.205
Appendectomy (n)	1/34	3/32	.614
Family history of UC, n (%)	0 (0%)	5 (14.3%)	.0536
Mesalazine (n)	–	23	–
Vedolizumab (n)	–	2	–
Azathioprine (n)	–	3	–
Rhofanib (tofacitinib) (n)	–	1	–
Adalimumab (n)	–	3	–
Infliximab (n)	–	1	–
Asacol (n)	–	1	–

BMI = body mass index, HDL‐C = high‐density lipoprotein cholesterol, LDL‐C = low‐density lipoprotein cholesterol, TG = triglycerides, UC = ulcerative colitis.

### 3.2. Tissue gene expression of PACER, MIAT, lincRNA-p21, and lincRNA-Cox2 in UC and control subjects

**Figure 1.**
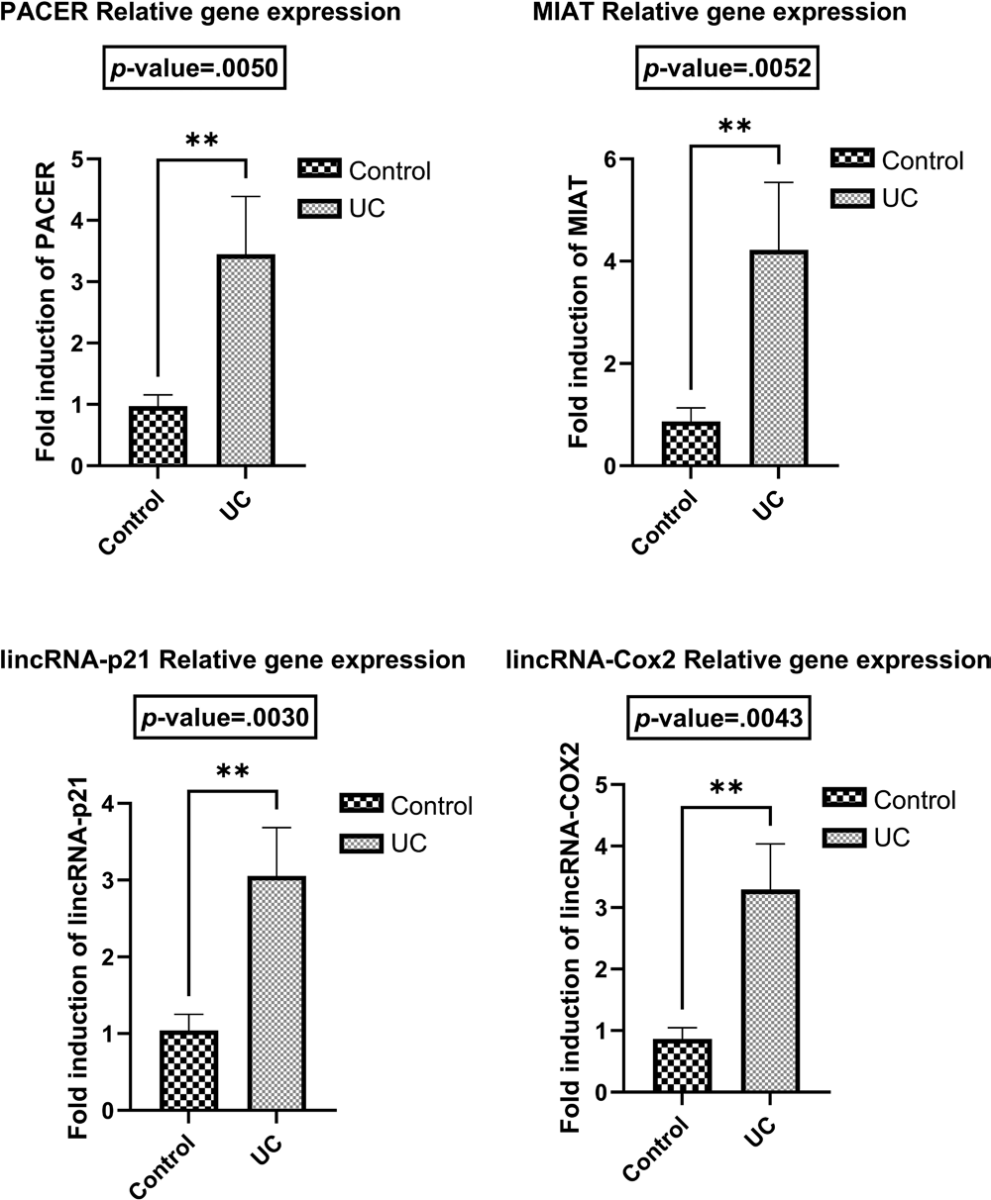
Level of PACER, MIAT, lincRNA-p21, and lincRNA-Cox2 in UC and control subjects. MIAT = myocardial infarction associated transcript, UC = ulcerative colitis. lincRNA-Cox2 = prostaglandin-endoperoxide synthase 2, opposite strand 2, MIAT = Homo sapiens myocardial infarction associated transcript, PACER = PTGS2 antisense NFKB1 complex-mediated expression regulator RNA.

This study found that the expression of PACER, MIAT, lincRNA-p21, and lincRNA-Cox2 was significantly upregulated in the colon tissues of patients with UC compared to healthy controls. The expression levels of PACER (*P* = .005), MIAT (*P* = .0052), lincRNA-p21 (*P* = .003), and lincRNA-Cox2 (*P* = .0043) were significantly higher in the UC group. To ensure accuracy, the relative expression levels of these genes were normalized to the expression of the 18s rRNA (Fig. 1).

### 3.3. Protein expression of NF-κB p65 and p-NF-κB p65

**Figure 2.**
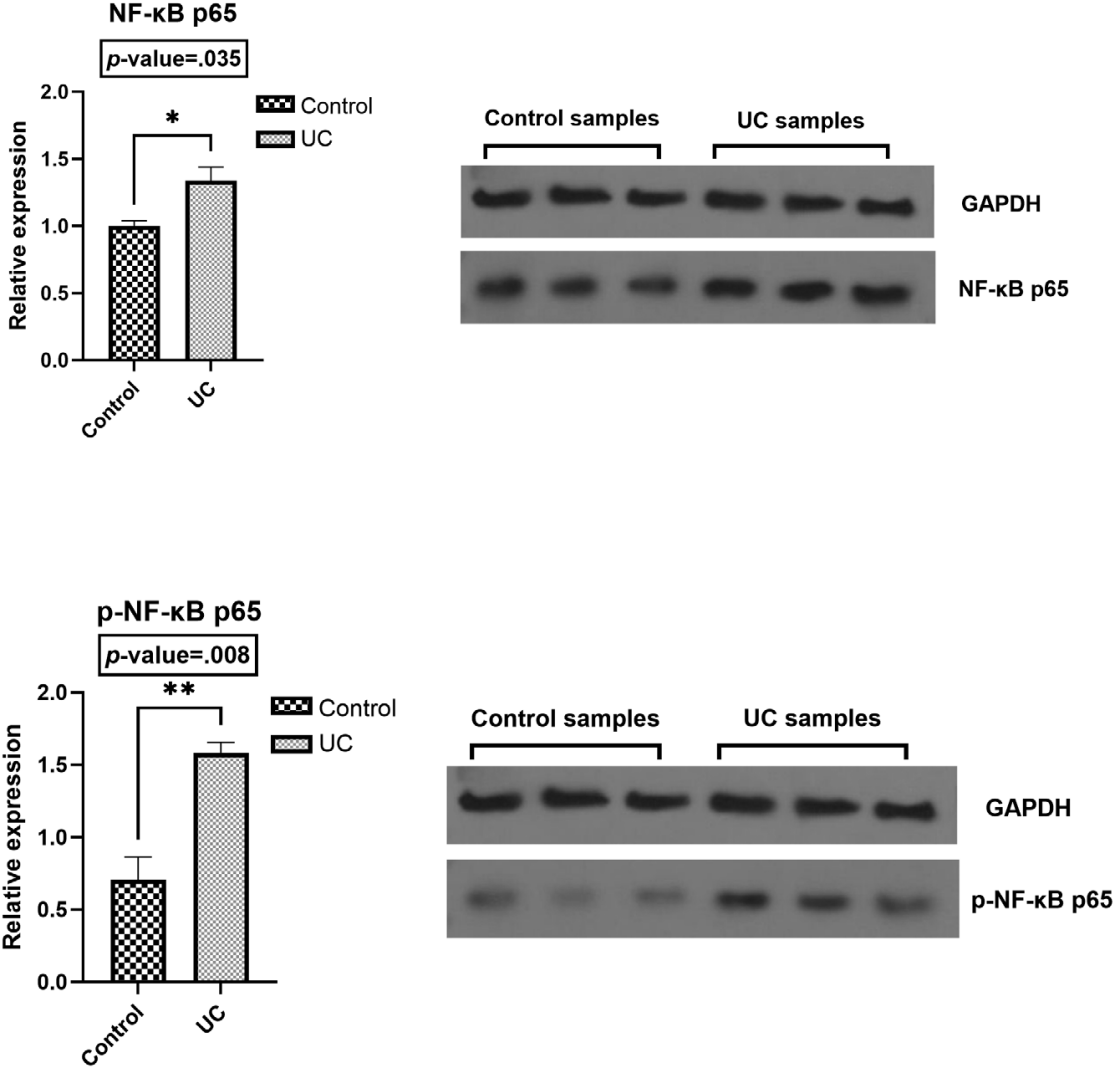
Protein level of NF-κB p65 and p-NF-κB p65 in UC and control subjects. The 2 groups’ protein expression was compared using appropriate statistical tests (unpaired *t*-test). UC = ulcerative colitis.

This study found that the levels of NF-κB p65 and p-NF-κB p65 were significantly elevated in the colon tissues of patients with UC compared to healthy controls (*P* = .035 and *P* = .008, respectively). Furthermore, the ratio of p-NF-κB p65 to NF-κB p65 was also significantly higher in the UC group (*P* = .0047). The upregulation of NF-κB p65 and p-NF-κB p65, along with the increased ratio of these proteins, indicates both increased expression and activity of NF-κB in the UC group. Protein levels were normalized to GAPDH expression, which served as a loading control (Fig. 2).

### 3.4. Correlation analysis of lncRNA expression

**Figure 3.**
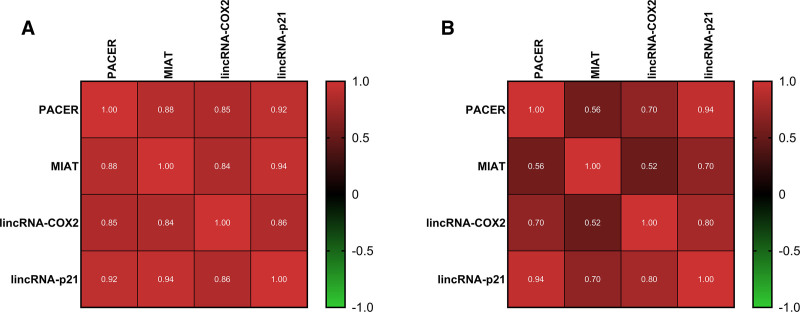
Pearson correlation heatmaps for the expression of PACER, MIAT, lincRNA-p21, and lincRNA-Cox2. The correlation coefficients (*r* values) are shown for (A) the UC patient samples and (B) the healthy control group samples. The color scale indicates the strength of the correlation from weak (darker colors) to strong (brighter colors). lincRNA-Cox2 = prostaglandin-endoperoxide synthase 2, opposite strand 2, MIAT = Homo sapiens myocardial infarction associated transcript, PACER = PTGS2 antisense NFKB1 complex-mediated expression regulator RNA, UC = ulcerative colitis.

To investigate the correlation between the expression of the 4 lncRNAs, Pearson correlation analysis was performed. In the UC patient group, a significant positive correlation was observed between all lncRNAs (*P* < .0001 for all pairs), with the specific correlation coefficients (r) shown in Figure 3A. A similar pattern was seen in the control group, where the correlations were also highly significant (*P* < .0001), with the exception of the correlations between MIAT and lincRNA-Cox2 (*P* = .005) and between PACER and lincRNA-Cox2 (*P* = .003). The corresponding correlation coefficients for the control group are presented in Figure 3B.

### 3.5. Correlation between lncRNA expression and biochemical and clinical parameters

To investigate the association between lncRNA expression and biochemical and clinical parameters, Pearson correlation analysis was used for data with a normal distribution, and Spearman correlation analysis was used for data with a non-normal distribution. In patients with UC, the expression of PACER, lincRNA-p21, and lincRNA-Cox2 was negatively correlated with BMI (r = -0.486, *P* = .006; r = -0.406, *P* = .024; and r = -0.444, *P* = .012, respectively). Additionally, the expression of PACER and lincRNA-Cox2 was negatively correlated with age (r = -0.373, *P* = .039 and r=-.358, *P* = .048, respectively). However, there were no significant correlations between lncRNA expression and any biochemical or clinical parameters in the control group.

### 3.6. Correlation between lncRNA expression and NF-κB activation

To investigate the association between the expression of the 4 lncRNAs and NF-κB activation in the UC group, Pearson correlation analysis was performed between the -ΔCt values of the lncRNAs and p-NF-κB p65 protein levels. A significant positive correlation was observed between the expression of PACER, MIAT, lincRNA-p21, and lincRNA-Cox2 and p-NF-κB p65 levels (*R* = 0.463, *P* = .01; *R* = 0.366, *P* = .047; *R* = 0.414, *P = *.023; and *R* = 0.387, *P* = .035, respectively).

### 3.7. Evaluation of diagnostic accuracy of lncRNAs using ROC curve analysis

**Figure 4.**
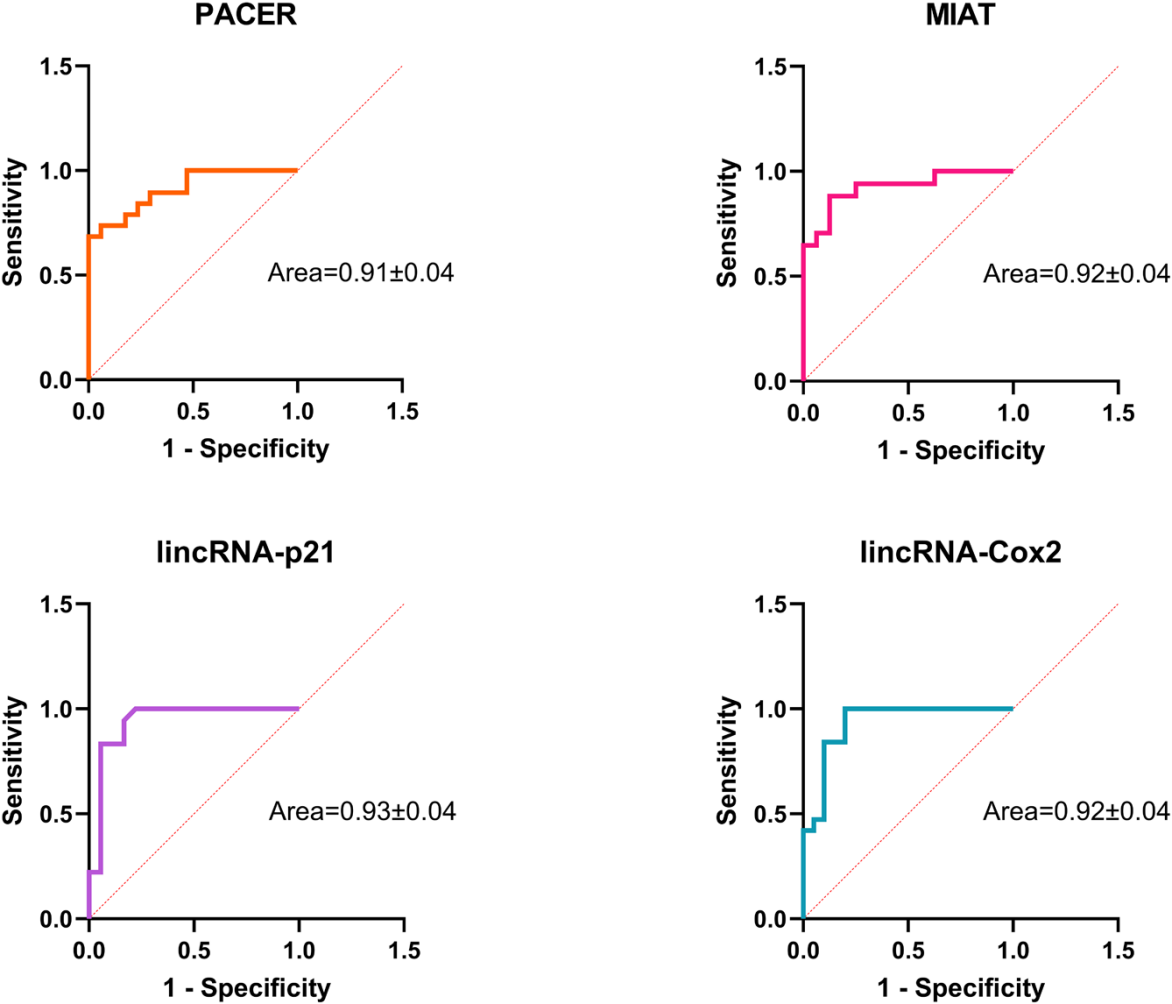
The area under the ROC curve (AUC) for PACER, MIAT, lincRNA-p21, and lincRNA-Cox2. AUC = area under the curve, lincRNA-Cox2 = prostaglandin-endoperoxide synthase 2, opposite strand 2, MIAT = Homo sapiens myocardial infarction associated transcript, PACER = PTGS2 antisense NFKB1 complex-mediated expression regulator RNA, ROC = receiver operating characteristic.

To evaluate the diagnostic accuracy of the 4 lncRNAs, ROC curve analysis was performed using the ΔCt values. The area under the ROC curve (AUC) for PACER, MIAT, lincRNA-p21, and lincRNA-Cox2 was 0.9102, 0.9228, 0.9367, and 0.9289, respectively (*P* < .0001 for all) (Fig. 4). These results suggest that these lncRNAs have good diagnostic potential for differentiating between individuals with UC and healthy controls. The sensitivity and specificity of each lncRNA are shown in Table 2.

**Table 2:** Sensitivity and specificity of PACER, MIAT, lincRNA-p21, and lincRNA-COX2 in ROC curve analysis.

Gene	Sensitivity	Specificity
PACER	73%	94%
MIAT	88%	87%
lincRNA-p21	94%	83%
lincRNA-Cox2	94%	80%

lincRNA-Cox2 = prostaglandin-endoperoxide synthase 2, opposite strand 2, MIAT = Homo sapiens myocardial infarction associated transcript, PACER = PTGS2 antisense NFKB1 complex-mediated expression regulator RNA, ROC = receiver operating characteristic.

## 4. Discussion

In the initial characterization of our study groups, we observed a significant difference in several biochemical parameters, most notably a decrease in TG levels in UC patients compared to healthy controls. This finding is particularly interesting when viewed in the context of the large-scale meta-analysis by Chen et al.,^[12]^ which investigated serum lipid profiles in inflammatory bowel disease patients. While their analysis did not find a statistically significant overall alteration in TG levels for the UC subgroup, they noted that results across individual studies are often heterogeneous. The mechanisms proposed for such lipid alterations are multifactorial, stemming from the complex interplay between systemic inflammation, which can affect hepatic lipid metabolism, and potential nutrient malabsorption due to intestinal mucosal damage. Therefore, our observation of lower TG levels could reflect the specific inflammatory and metabolic state of our patient population.

lncRNAs have emerged as critical regulators of gene expression and various cellular processes. Dysregulation of lncRNA expression has been implicated in a wide range of diseases, particularly in inflammatory disorders such as UC. For example, Lin et al.^[22]^ reported that the lncRNA DLEU2 is upregulated in UC and is negatively associated with the expression of inflammatory cytokines such as TNF-α, IL-1α, IL-1β, and IL-6. DLEU2 also inhibits the NF-κB pathway, exerts anti-inflammatory effects, and may serve as a potential diagnostic biomarker and therapeutic target. Similarly, Zhu et al.^[23]^ found that the lncRNA MALAT1 is increased in the tissue and serum of UC patients and promotes disease progression by increasing the expression of the lncRNA ANRIL. Pan et al.^[24]^ reported that the lncRNA NEAT1 is upregulated in UC and activates the NF-κB pathway by increasing the expression of TNFRSF1B and promoting the translocation of p65, leading to increased inflammation. In this study, we investigated the expression of 4 lncRNAs – PACER, MIAT, lincRNA-p21, and lincRNA-Cox2–in the colon tissues of patients with UC compared to healthy controls. We also explored the potential association between these lncRNAs and the activation of the NF-κB signaling pathway, a key regulator of inflammation.

Our results demonstrate that the expression of PACER, MIAT, lincRNA-p21, and lincRNA-Cox2 is significantly upregulated in the colon tissues of patients with UC compared to healthy controls. The increased expression of PACER, MIAT, and lincRNA-CoX2 is consistent with previous studies reporting their upregulation in other inflammatory diseases. For example, Desind et al.^[25]^ showed that PACER expression is increased in lung cancer cells and promotes inflammation by increasing the expression of Cox2. Similarly, Attia et al.^[26]^ demonstrated that lincRNA-Cox2 expression is elevated in patients with relapsing-remitting multiple sclerosis and is positively correlated with the expression of inflammatory cytokines such as IL-1β. Xu et al.^[27]^ also reported increased expression of MIAT in gastric cancer, which was associated with poor prognosis. In contrast to the consistent findings for PACER, MIAT, and lincRNA-Cox2, the upregulation of lincRNA-p21 observed in our study appears to be in conflict with some previous reports. For instance, Chen et al.^[28]^ and Yang et al.^[29]^ reported that lincRNA-p21 functions as a tumor suppressor and is downregulated in gastric cancer and hepatocellular carcinoma, respectively. However, our findings are consistent with a study by Zhang et al.,^[30]^ which showed that lincRNA-p21 expression is elevated in acute respiratory distress syndrome.

One of the hallmarks of UC is chronic inflammation in the intestinal mucosa. Numerous factors contribute to this inflammatory process, including the activation of the NF-κB signaling pathway. NF-κB is a key regulator of the inflammatory response and has been extensively studied as a potential therapeutic target in UC. Several studies have demonstrated that NF-κB activation is increased in the inflamed mucosa of UC patients.

In this study, we observed a significant increase in the levels of NF-κB p65 and p-NF-κB p65 in the colon tissues of UC patients compared to healthy controls, indicating increased expression and activity of NF-κB. Furthermore, we found a positive correlation between the expression of PACER, MIAT, lincRNA-p21, and lincRNA-Cox2 and the levels of p-NF-κB p65, suggesting that these lncRNAs may be associated in the activation of the NF-κB pathway in UC. The precise mechanisms by which these lncRNAs regulate NF-κB signaling are not yet fully understood. Some studies have suggested that PACER activates NF-κB by binding to the p50 subunit reducing the formation of p50 homodimers and promoting the formation of p50/p65 heterodimers, which are capable of activating transcription. Thus, PACER can activate NF-κB by binding to p50 and modulating the composition of NF-κB dimers, but it does not directly interact with or modify p65[19]. However, other studies have reported that NF-κB can directly regulate PACER expression, suggesting a complex interplay between this lncRNA and the NF-κB pathway.^[25]^ Regarding MIAT, its association with NF-κB activation has also been described. For instance, MIAT has been reported to promote activation of the TRAF6/NF-κB signaling axis by functioning as a competing endogenous RNA for miR-330-5p.^[31]^ In line with a role in promoting NF-κB activity, studies have shown that inhibiting MIAT can lead to reduced p65 nuclear translocation and decreased NF-κB activity.^[32]^ Additionally, lincRNA-Cox2 and lincRNA-p21 have been implicated in NF-κB activation by promoting the translocation of p65 to the nucleus.^[30,33]^ Further research is needed to fully elucidate the interplay between these lncRNAs and the NF-κB signaling pathway in the pathogenesis of UC.

ROC curve analysis revealed that PACER, MIAT, lincRNA-p21, and lincRNA-Cox2 have good diagnostic potential for differentiating between individuals with UC and healthy controls. These findings suggest that these lncRNAs may serve as novel biomarkers for the diagnosis of UC. However, further studies with larger cohorts are needed to validate these findings and assess the clinical utility of these lncRNAs in the diagnosis and prognosis of UC.

To translate this potential into a clinical context, a closer analysis of the individual markers is warranted. Among them, lincRNA-p21 showed the highest individual performance with an AUC of 0.9367. However, a diagnostic panel is often more robust for clinical use. A combination approach, potentially leveraging the high sensitivity of markers like lincRNA-p21 (94%) with the high specificity of PACER (94%), could provide a more balanced and powerful diagnostic tool. It is crucial to emphasize that these are preliminary results. Extensive validation in larger, multi-center cohorts is an essential next step before these lncRNAs can be considered for any clinical or commercial application.

This study has several limitations that should be acknowledged, primarily related to baseline differences between the study groups. A notable limitation is the significant difference in age and BMI. The lower median age in our UC population is consistent with the established epidemiology of the disease,^[3]^ while the lower BMI aligns with findings from large cohort studies, such as the one by Mendall et al.,^[34]^ which reported an inverse association with UC risk. Concurrently, another key limitation is the heterogeneity of medication use within the UC group. As these various therapies are known to modulate inflammatory pathways, we cannot exclude their potential confounding effects on the observed lncRNA expression levels. Interestingly, our own correlation analysis revealed a negative relationship between age, BMI, and the expression of several lncRNAs. While this may suggest our main finding of upregulation is robust despite these confounders, future studies using larger, age- and BMI-matched cohorts that also allow for stratification by medication type are warranted to validate these findings.

In conclusion, our study demonstrates that the expression of PACER, MIAT, lincRNA-p21, and lincRNA-Cox2 is dysregulated in the colon tissues of patients with UC. These findings suggest that these lncRNAs may play a role in the pathogenesis of UC, and that altered expression of these lncRNAs may be associated with the activation of the NF-κB signaling pathway. Further studies are needed to elucidate the precise mechanisms by which these lncRNAs contribute to UC pathogenesis and to explore their potential as therapeutic targets. Additionally, our results suggest that these lncRNAs may serve as potential biomarkers for the diagnosis of UC. However, validation through further research with larger cohorts is essential to assess their clinical utility.

## 5. Conclusion

In this study, we investigated the expression of 4 lncRNAs – PACER, MIAT, lincRNA-p21, and lincRNA-Cox2–in the colon tissues of patients with UC compared to healthy controls. Our results demonstrate that the expression of these lncRNAs is significantly upregulated in UC patients. Furthermore, we observed increased NF-κB activity in the UC group, and a positive correlation was found between lncRNA expression and NF-κB activation. However, further studies are needed to elucidate the precise role of these lncRNAs in the regulation of NF-κB signaling. Our findings suggest that these lncRNAs may serve as potential diagnostic biomarkers for UC, but further research with larger cohorts is needed to validate these findings and assess their clinical utility.

## Acknowledgments

We express our deepest appreciation to the staff of the Gastroenterology and Liver Clinic at Shariati Hospital in Tehran for their exceptional assistance and dedication to this research. We are also incredibly grateful to all the participants for their invaluable contribution to this study.

## Author contributions

**Data curation:** Amin Karimpour.

**Formal analysis:** Amin Karimpour, Amir Anoshiravani, Farzad Farzamifar.

**Writing – original draft:** Amin Karimpour.

**Writing – review & editing:** Amir Anoshiravani, Maryam Jabarpour, Farzad Farzamifar, Ghodratollah Panahi.

**Methodology:** Mina Zare.

**Conceptualization:** Ghodratollah Panahi.

**Funding acquisition:** Ghodratollah Panahi.

**Investigation:** Ghodratollah Panahi.

**Project administration:** Ghodratollah Panahi.

**Supervision:** Ghodratollah Panahi.
